# Neuroendocrine transformation of prostate adenocarcinoma with gallbladder metastases: Unusual case

**DOI:** 10.1016/j.radcr.2025.11.005

**Published:** 2025-12-04

**Authors:** Fatima Safini, Imane Slimani, Achraf Miry, Soufiane Bigi, Soukaina Wakrim, Sanae Abbaoui, Bouchra Amaoui

**Affiliations:** a Biotechnology and Medicine (BioMed) Laboratory, Faculty of Medicine and Pharmacy, Ibn Zohr University, Agadir, Morocco; b Department of Radiotherapy, Souss-Massa University Hospital Center, Agadir, Morocco; c Department of Pathology and Molecular Biology, Souss-Massa University Hospital Center, Agadir, Morocco; d Department of Radiology, Souss-Massa University Hospital Center, Agadir, Morocco

**Keywords:** Neuroendocrine transformation, Prostate cancer, Gallbladder, Metastases

## Abstract

Gallbladder metastasis from prostate cancer is exceptionally rare. We report the case of a 66-year-old man initially treated for high-risk prostatic adenocarcinoma with a combination of external beam radiotherapy and 2 years of androgen deprivation therapy. Six months after discontinuation of hormonal treatment, the patient developed bone and gallbladder metastases. Histopathological examination of the cholecystectomy specimen confirmed gallbladder involvement secondary to neuroendocrine transformation of the primary prostate cancer. To our knowledge, this is the first North African case of gallbladder metastasis arising from a neuroendocrine-transformed prostatic adenocarcinoma. We describe the clinical presentation and provide a brief literature review.

## Introduction

Prostate cancer remains one of the most prevalent malignancies among men worldwide, most often presenting with bone, lymph node, or visceral metastases [1]. Metastatic spread to the gallbladder, however, is exceedingly uncommon. Neuroendocrine differentiation represents a rare and aggressive evolution of prostate adenocarcinoma, typically arising in the context of castration-resistant disease and associated with poor prognosis [2]. We report a new and exceptional case of neuroendocrine-transformed prostate cancer with gallbladder metastasis that expands the spectrum of atypical metastatic sites associated with prostate cancer.

## Case report

We report the case of a 66-year-old man previously treated in 2008 for a non-muscle-invasive bladder tumor. He underwent transurethral resection followed by intravesical BCG therapy.

In 2019, the patient presented with lower urinary tract symptoms, including frequency, dysuria, and burning micturition. Clinical examination revealed an indurated prostate with a firm nodule in the right lobe. His serum PSA level was 19 ng/mL.

**Fig. 1:**
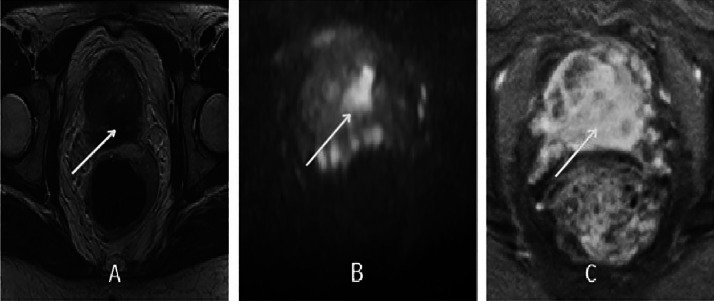
Axial-section MRI images in T2 (A), Diffusion (B) and T1 sequence after gadolinium injection (C), showing a tumor patch in the medio-prostatic peripheral zone in T2 hyposignal and Diffusion hyper signal with significant gadolinium uptake after injection (white arrows).

A prostate biopsy confirmed adenocarcinoma involving both lobes, with a Gleason score of 9 (4 + 5), WHO/ISUP grade group 5, and evidence of perineural invasion. Prostate MRI demonstrated a suspicious 17 mm lesion with capsular breach and invasion of the seminal vesicles and posterior bladder wall (Fig. 1). The staging workup—including thoraco-abdominal CT and bone scintigraphy—showed no metastatic lesions. After discussion in a multidisciplinary tumor board, the patient received external beam radiotherapy combined with androgen deprivation therapy using an LHRH analog for 2 years.

**Fig. 2:**
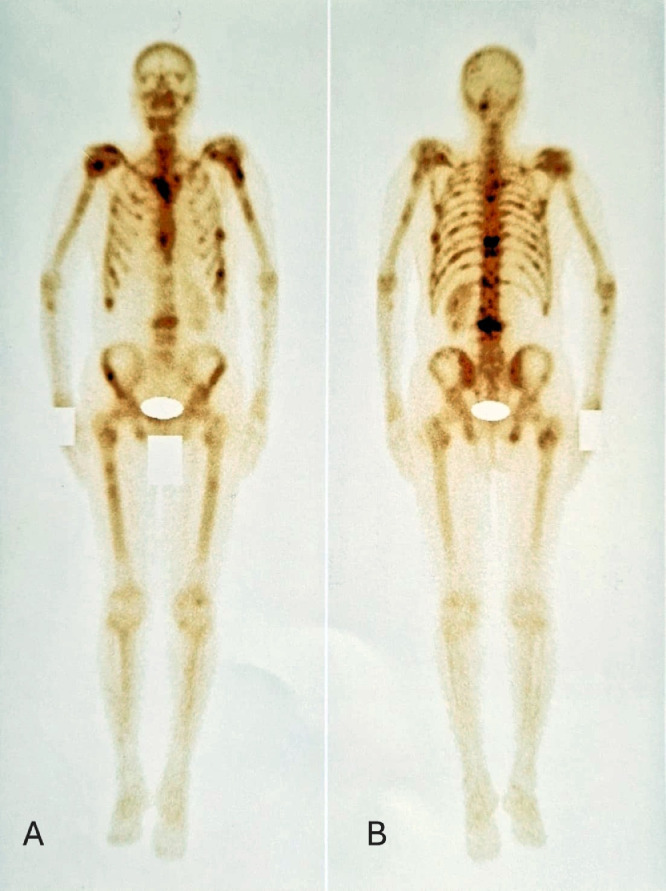
Whole-body bone scintigraphy showing anterior (A) and posterior (B) projections with multiple foci of increased tracer uptake consistent with bone metastases.

**Fig. 3:**
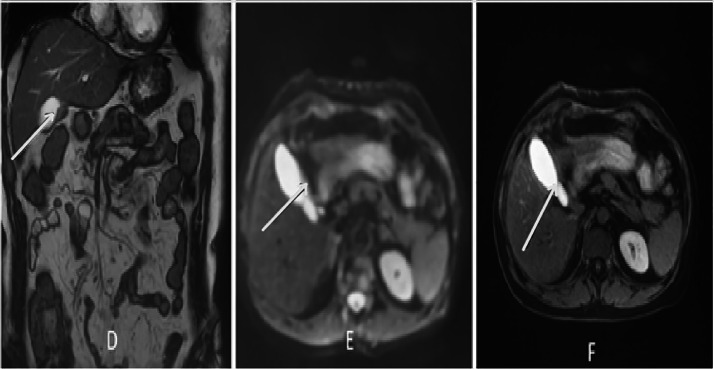
MRI images in coronal T2 section (D), DWI axial section (E) and T1 axial section after gadolinium injection (F), showing a parietal lesion of the gallbladder, protruding endoluminal, in T2 and DWI hyper signal, taking up gadolinium after injection (white arrows).

**Fig. 4:**
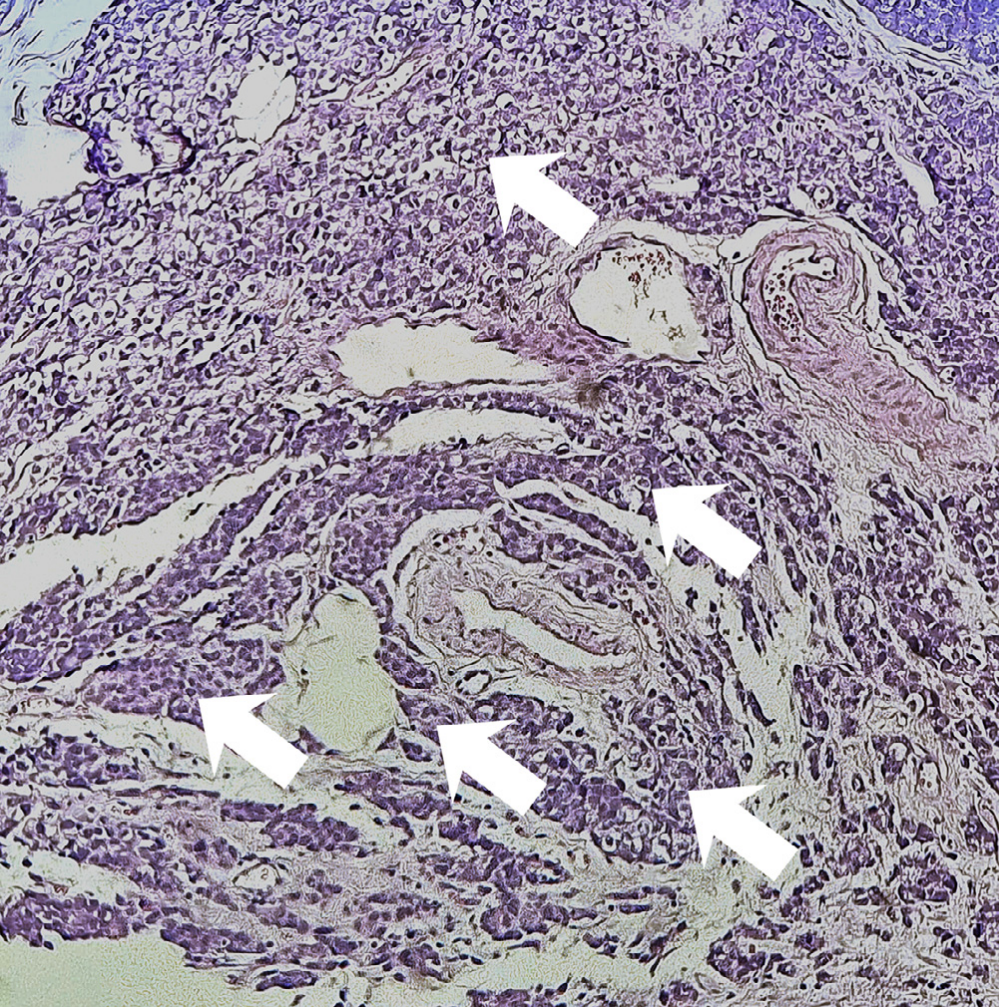
Microphotograph showing diffuse infiltration (white arrows) of the gallbladder wall.

**Fig. 5:**
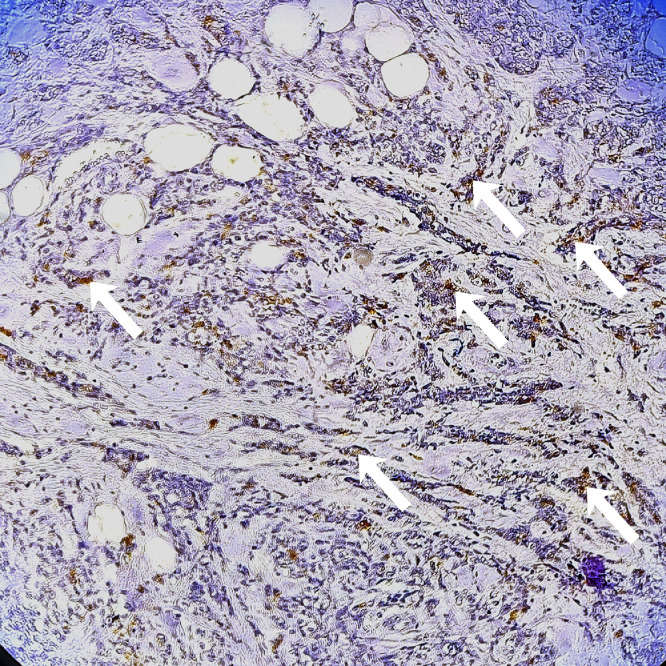
Tumor cells demonstrating positive immunostaining for PSA antigen (white arrows).

**Fig. 6:**
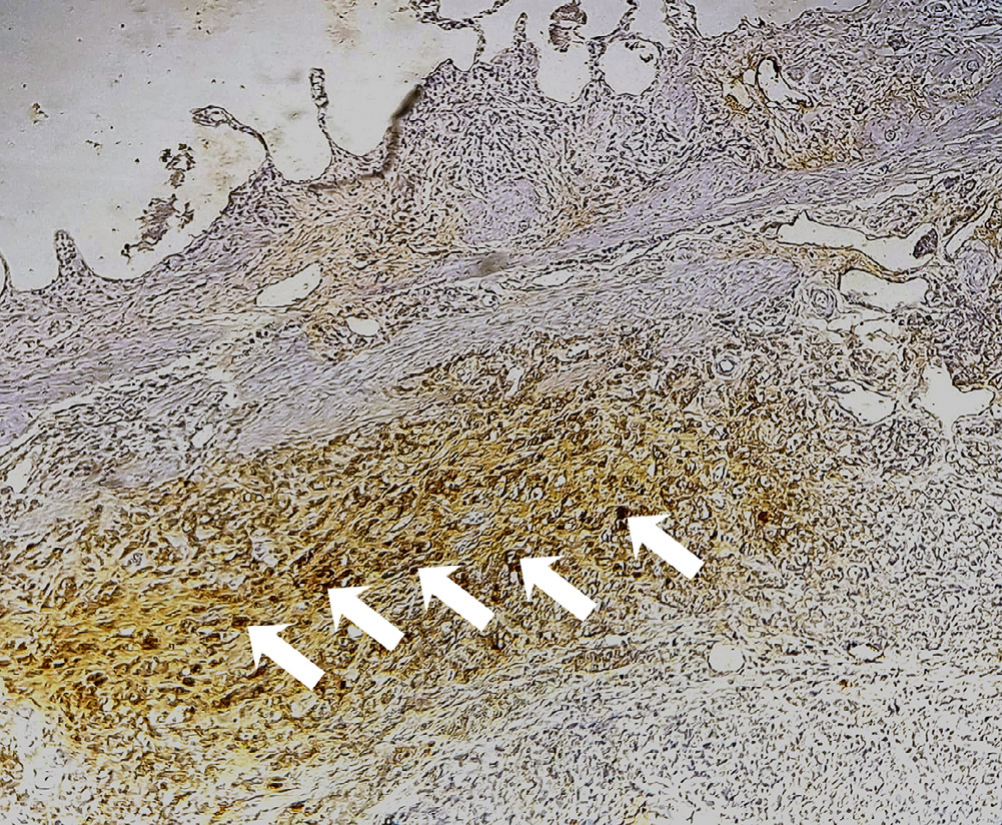
Tumor cells demonstrating positive immunostaining for chromogranin A antigen (white arrows).

Six months after discontinuation of hormonal therapy, he developed severe lumbosacral pain refractory to analgesics. PSA had increased to 5.43 ng/mL. Bone scintigraphy showed intense and diffuse axial and peripheral skeletal metastases radiotracer uptake. Palliative radiotherapy was delivered to vertebra L3 at a total dose of 30 Gy in 10 fractions, followed by bisphosphonate therapy and treatment with abiraterone acetate. The patient later complained of right upper quadrant pain. Thoraco-abdomino-pelvic CT revealed, in addition to bone lesions, marked thickening of the gallbladder wall. Magnetic resonance imaging (MRI) of the liver revealed a gallbladder wall lesion protruding into the lumen and enhancing after gadolinium injection (Fig. 2). Laparoscopic cholecystectomy was performed. Macroscopic examination of the specimen showed a fleshy, tumorous thickening of the gallbladder wall located 1 cm from the cystic duct margin. Microscopic examination revealed massive infiltration of the submucosa and serosa by poorly differentiated carcinoma cells (Fig. 3, Fig. 4). Immunohistochemical staining showed diffuse tumor positivity for cytokeratin (AE1/AE3) and chromogranin A (Fig. 5), with focal expression of PSA (Fig. 6) and synaptophysin. These findings confirmed the diagnosis of gallbladder metastasis from prostatic carcinoma with neuroendocrine differentiation.

The patient subsequently received 6 cycles of combination chemotherapy with etoposide and cisplatin. The clinical course was complicated by pancytopenia, and bone marrow biopsy demonstrated massive metastatic infiltration by prostate carcinoma. The patient died 10 months after gallbladder surgery.

## Discussion

Prostate cancer is the most common malignancy among elderly men worldwide, with an estimated 1.3 million new cases annually, and projections reaching 2.3 million by 2040 [1]. The emergence of a neuroendocrine component may occur during the natural evolution of prostatic adenocarcinoma, particularly at the stage of castration resistance. This transformation reflects tumor aggressiveness and the development of resistance to androgen receptor–targeted therapies [2]. In our patient, neuroendocrine transformation occurred during disease progression. Chemotherapy is typically indicated in such cases, drawing parallels with pulmonary neuroendocrine carcinomas, with first-line treatment generally based on the etoposide–cisplatin regimen [3]. However, genomic profiling is increasingly recommended to guide personalized targeted therapy.

Gallbladder metastases are rare, accounting for approximately 4.8% of all malignant gallbladder tumors, and usually result from hematogenous dissemination. Nevertheless, direct invasion from adjacent intra-abdominal malignancies such as colorectal or gastric cancer must first be excluded [4]. These metastases are often diagnosed at an advanced stage or incidentally at autopsy. The most frequent primary source is melanoma (55.6%), followed by breast carcinoma (13.6%), hepatocellular carcinoma (13.6%), and renal cell carcinoma (6.8%) [5].

Bone remains the most common metastatic site for prostate cancer, followed by lung and liver. Other atypical metastatic sites have been reported, but gallbladder metastases originating from prostate carcinoma are exceptionally rare. In a review, Badrane et al. analyzed unusual metastatic sites of prostate cancer and reported no cases involving the gallbladder [6]. Similarly, Bubendorf et al. examined 19,316 autopsies of men over 40 years of age, identifying 1,589 cases (8.2%) of prostate carcinoma. Hematogenous metastases were present in 35% of these cases, most frequently involving bone (90%), lung (46%), and liver (25%), whereas gallbladder involvement was identified in only 0.5% [7]. Only a few cases have been described in the literature. The first was published by Maxwell et al. in 2009, followed by Kent et al. in 2014 [8,9] (Table 1). With the increasing use of sensitive imaging modalities such as ^18^F-PSMA PET/CT, the detection rate of rare metastatic sites is expected to rise [10].

**Table 1:** Clinical cases reported in the literature regarding gallbladder metastases from prostate cancer.

Study	Pub year	age	Clinical presentation	Histology	Gleason	Other metastases	treatment
Maxwell et al. [8]	2009	83	Acute cholecystitis	Prostatic adenocarcinoma	8	Isolated gallbladder metastase	Surgery: cholecystectomy + Bicalutamide, leuprolide and denosumab.
Kent et al. [9]	2014	72	Weight loss, nausea	Prostatic adenocarcinoma	9 (4 + 5)	bone	Surgery: laparoscopic cholecystectomy
Our case	2025	66	Abdominal pain	Mixte: Adenocarcinoma+ neuroendocrine carcinoma	9 (4 + 5)	Bone + bone marrow infiltration	Surgery: cholecystectomy + chemotherapy (etoposide/cisplatin)

Comprehensive management should adhere to current international guidelines for metastatic prostate cancer, which remain primarily based on systemic therapies. Local treatment of gallbladder lesions may be considered in selected oligo-metastatic patients with good performance status. Surgical resection can be discussed in multidisciplinary tumor board meetings for patients with isolated and resectable biliary metastases to prevent complications or symptomatic progression. Laparoscopic cholecystectomy is the preferred approach, emphasizing careful manipulation to avoid perforation. The use of an extraction bag minimizes the risk of tumor seeding. The standard surgical procedure consists of simple cholecystectomy without lymphadenectomy [11]. In a Korean series by Yoon et al., complete resection of macroscopic disease was associated with improved survival outcomes [12]. Median overall survival for patients with gallbladder metastases, regardless of the primary site, has been reported at 8.7 months. This poor prognosis reflects the typically late diagnosis and the frequent presence of widespread metastatic disease at the terminal stage, as observed in our patient [5].

## Conclusion

The early neuroendocrine differentiation observed during the progression of prostatic adenocarcinoma, together with the occurrence of gallbladder metastasis, highlights the need for further investigation into the molecular and cellular signaling pathways driving disease progression. Comprehensive genomic profiling in such cases is essential to guide the selection of targeted and effective therapeutic strategies.

## Patient consent

I would like to inform you that a written informed consent was obtained from the patient for publication of this case report and any accompanying images or data. The patient was informed that all personal identifying information would be removed to ensure anonymity. A copy of the signed consent form is retained by the authors and is available for review by the journal if requested.
